# Investigation of the antimicrobial activity and hematological pattern of nano-chitosan and its nano-copper composite

**DOI:** 10.1038/s41598-021-88907-z

**Published:** 2021-05-05

**Authors:** Somia B. Ahmed, Hadeer I. Mohamed, Abeer M. Al-Subaie, Ahoud I. Al-Ohali, Nesrine M. R. Mahmoud

**Affiliations:** 1 Department of Basic Sciences, Deanship of Preparatory Year and Supporting Studies, Imam Abdulrahman Bin Faisal University, P.O. Box 1982, Dammam, 34212 Saudi Arabia; 2 Department of Neuroscience Technology, College of Applied Medical Sciences in Jubial, Imam Abdulrahman Bin Faisal University, P.O. Box 4030, Jubail, 35816 Saudi Arabia; 3 Department of Clinical Laboratory Sciences, College of Applied Medical Sciences, Imam Abdulrahman Bin Faisal University, Dammam, Saudi Arabia

**Keywords:** Chemical biology, Chemistry, Materials science, Nanoscience and technology

## Abstract

Novel synthesized Chitosan–Copper oxide nanocomposite (Cs–CuO) was prepared using pomegranate peels extract as green precipitating agents to improve the biological activity of Cs-NP's, which was synthesized through the ionic gelation method. The characterization of biogenic nanoparticles Cs-NP's and Cs–CuO-NP's was investigated structurally, morphologically to determine all the significant characters of those nanoparticles. Antimicrobial activity was tested for both Cs-NP's and Cs–CuO-NP's via minimum inhibition concentration and zone analysis against fungus, gram-positive and gram-negative. The antimicrobial test results showed high sensitivity of Cs–CuO-NP's to all microorganisms tested in a concentration less than 20,000 mg/L, while the sensitivity of Cs-NP's against all microorganisms under the test started from a concentration of 20,000–40,000 mg/L except for the *C. albicans* species. The hematological activity was also tested via measuring the RBCs, platelet count, and clotting time against healthy, diabetic, and hypercholesteremia blood samples. The measurement showed a decrease in RBCs and platelet count by adding Cs-NP’s or Cs–CuO-NP's to the three blood samples. Cs-NP's success in decreasing the clotting time for healthy and diabetic blood acting as a procoagulant agent while adding biogenic CuO-NP’s to Cs-NP’s increased clotting time considering as an anti-coagulant agent for hypercholesteremia blood samples.

## Introduction

Throughout history, wound healing has been a crucial challenge facing all wound care researchers in the medical field. Wound healing is a process of repairing tissue integrity through a series of phases, including hemostasis, inflammation, proliferation and remodeling^1^. The initial stage, which is the initiation of a coagulation cascade to prevent excess blood loss leading to platelet accumulation and fibrin clot formation, is known as Haemostasis. Halting in any healing stage leads to chronic wounds susceptible to increased microbial infections, extensive exudates, and necrosis in tissues due to an upsurge in pus cells number^2,3^. Therefore, recent research has focused on improving wound dressing materials, specifically, those originated from natural polymers to becoming interactive and bioactive materials.

Chitosan is the most naturally abundant biopolymer and the second most abundant polymer after cellulose^4,5^. Chitosan being polycationic at acidic media (pH < 6) allows it to interact easily with negatively charged molecules, such as phospholipids, anionic polysaccharides, proteins, and fatty acids. Nonetheless, Chitosan may also chelate metal ions selectively, such as copper, iron, cadmium, and magnesium^6^. Chitosan plays a vital role in the regeneration of the wounded area via fibroblast proliferation and glucosamine presence, enhancing the earlier synthesis of hyaluronic acid to accelerate the healing process with minimal scarring^7^. It also helps in revascularization and plays a role in protecting against atherosclerosis^8^. Additionally, Chitosan can control inflammatory mediators to accelerate the healing process^8^.

Chitosan-based nanoparticles, being versatile, non-toxic, biocompatible, and biodegradable, snatched researchers' attention in the biomedical field^9,10^. Interests in improving nano chitosan properties via chemical modification have been overgrowing^10^. Chitosan chemical modification is of great interest because it can retain its basic skeleton, which keeps its physicochemical and biological properties^9^.

Chitosan with various modifications^7,8^, and several reactive functional sites has shown high activity as an innate antibacterial agent, especially when mixed with metallic nanoparticles. Copper is one of the metallic nanoparticles, which is a vital element, in trace amounts, that facilitates various enzymes, and it also helps in skin regeneration, wound healing process, and angiogenesis^11^. Some previous studies showed restraints concerning Copper due to its toxicity, which is known to emerge from the production of oxy-radicals, which initiates ROS formation, resulting in oxidative stress^11,12^. However, the literature revealed that the hybridization of Copper with Chitosan reduces the toxicity level^13^. Also, it was reported that nano Cu and CuO are considered effective antibacterial agents^13–15^. According to the literature, the chemical reduction method is a facile process for synthesizing NP’s using biopolymeric materials^16^ in achieving a better substantial bacteriostatic/bactericidal property.

Copper/Copper oxide nanoparticles (Cu/CuO-NP’s) were biologically synthesized using different plant extracts as reducing agents as well as capping agents. These plant extracts have promising advantages for enhancing the biological activity of the CuO-NP’s. Pomegranate peel is rich with significant amounts of polyphenols, that is, phenolic acids, such as ellagic and gallic acid, flavonoids, and Tannis^17,18^, which are effective as antimicrobials, antianxiety, antidepressant, antiproliferative, antitumor, antioxidant agents^19,20^ anti-coagulants, antiplatelets, and anti-anemic agents^21,22^ and play a preventive role in cardiovascular diseases by inhibiting coagulation and thrombus^23^. Also, it was proved that it had a vital role in treating the blood vessels and heart, such as heart attack, atherosclerosis, and high cholesterol. It is also used for conditions of some digestive tract diseases, including diarrhea and intestinal parasites^24^. Anti-coagulant, antiplatelet, and hypofibrinogenemic effects of *P. granatum* may be due to thrombin's impaired activity predominantly by TAT complex and PC^25^.

This work aims to synthesize a hybrid bioactive nanocomposite from chitosan co-polymer, which provides antibacterial efficiency, playing a pivotal role in the healing process.

## Results and discussion

### Structural and morphological characterization

**Figure 1:**
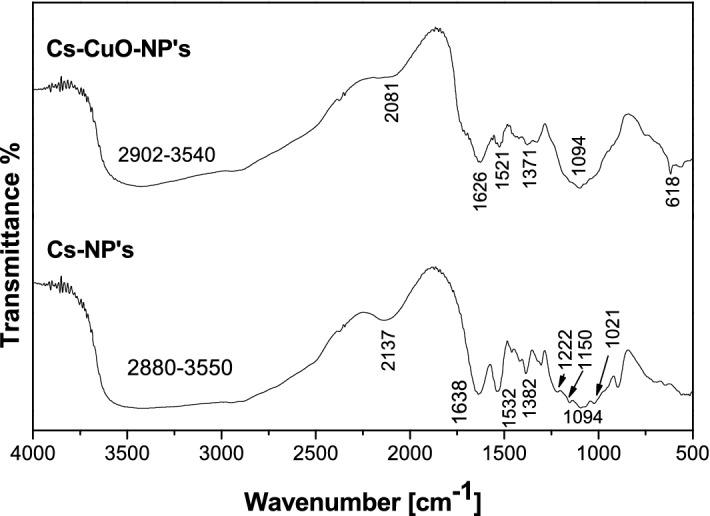
FTIR for **(a)** Cs-NP's, **(b)** Cs–CuO-NP's nanocomposite.

FT-IR of biogenic synthesized Cs-NP’s and Cs–CuO-nanocomposite was investigated (Fig. 1) and the characteristic spectrum pattern summarized as following, the significant Cs-NP’s bands were found at 3550–3000 cm^−1^ for overlapping between N–H and –OH stretching^26,27^. The band at 1638 cm^−1^ indicates the presence of cm^−1^ carbonyl group of amides belong to non-deacetylated part of Chitosan^27^. Band at 1532 cm^−1^ for N–H bending vibration of the amide group^27^. At 1382 cm^−1^ band belong to CH_3_ symmetrical deformation mode in amide group^26^. The band of stretching vibration of C–O–C linkages of polysaccharides^27^ found at 1100 cm^−1^. At 1094 and 1021 cm^−1^, bands represent skeletal stretching vibration of C–O^26^. Band of P=O stretching^27^ found at 1222 cm^−1^. While the significant bands of hybrid Cs–CuO-nanocomposite were found at 3550–2902 cm^−1^ indicate the overlapping between N–H, –OH stretching from Chitosan^26^with –OH of carboxylic acid, phenol or alcohol and C–H stretching vibration of aliphatic compound of pomegranate peel^28^. Many bands corresponded to the chitosan part at 1626, 1532 and 1371 cm^−1^ representing the existence of carbonyl group, N–H bending vibration and CH_3_ symmetrical deformation mode^27^ ,respectively. The band at 1327 cm^−1^ indicates the skeletal vibration of the aromatic ring of pomegranate peel^28^. Skeletal stretching vibration of C–O^27^ of both chitosan and pomegranate peel found at 1094 cm^−1^.On the other hand, the appearance of a band at 616 cm^−1^ and the shifting in many bands' locations refer to the interaction between nano chitosan and CuO^26–29^. Finally, the above data reveals the formation of hybrid nanocomposite from nano-chitosan and copper oxide, capped by pomegranate peel extract.

**Figure 2:**
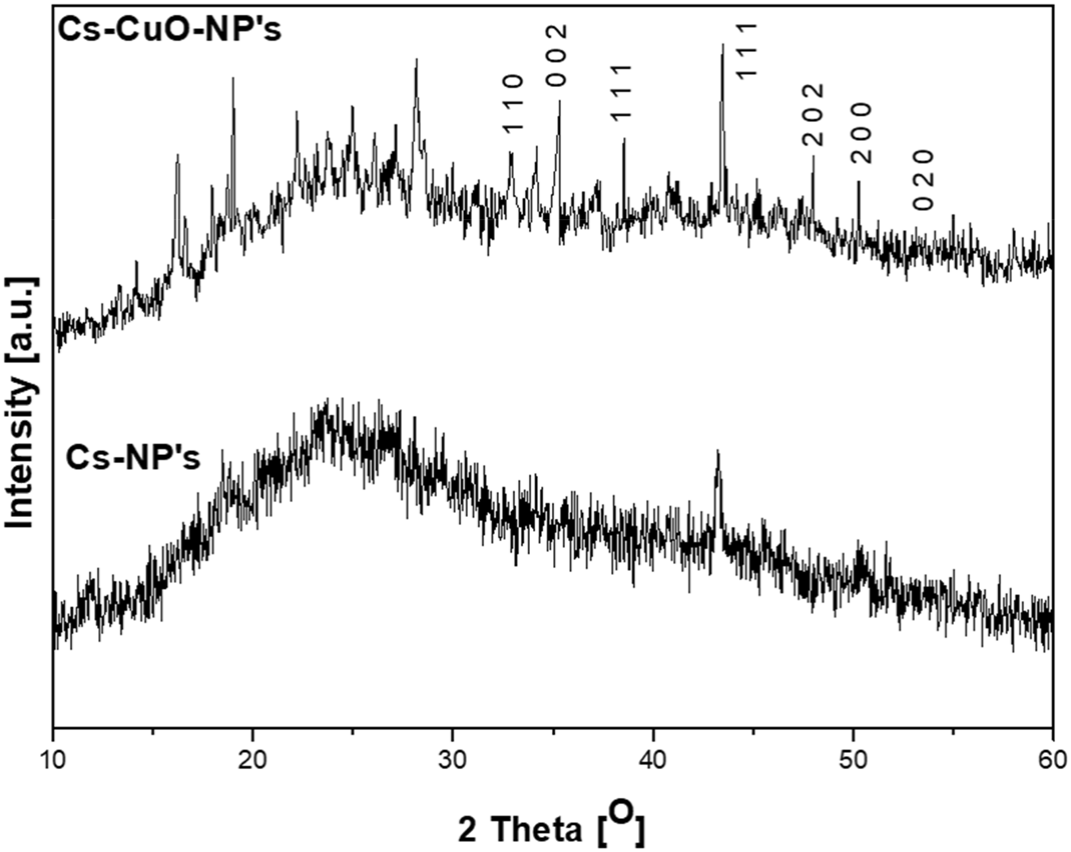
XRD patterns for **(a)** Cs-NP's, **(b)** Cs–CuO-NP's.

XRD analysis describes the crystalline structure and assesses the compatibility of each component present in the synthesized composite. Figure 2 shows the XRD patterns of Cs-NP’s and Cs–CuO-NP’s. The XRD of chitosan nanoparticles (Cs-NP's) had broadband at 2θ = 25° due to the crystalline regions' deformation, which led to ionic crosslinking with tripolyphosphate, increasing the disarray of Chitosan chains resulting in the formation of amorphous chitosan nanoparticles^30^. This could be ascribed as a result of the substitution of hydroxyl and amino groups due to the deformation of the hydrogen bond in the original chitosan chain^31^, which efficiently breakdown the regularity of the leading Chitosan chains packing.

Cs–CuO-NP’s show crystalline peaks of mixed phases of CuO and metallic Cu. CuO patterns were recorded at 2θ = 32.7°, 35.3°, 38.7°, 48.0°, and 53.2° which was assigned to (− 110), (002), (111), (− 202), and (020) reflections, respectively^32^ of the monoclinic structure of the CuO phase, in agreement with JCPDS card No. 45–0937 with lattice parameters a = 0.4685 nm, b = 0.3889 nm, and c = 0.513 nm, along with angles α = γ = 90° and β = 99.549°. Cu patterns were detected at 2θ = 43.6° and 50.3° which were assigned to (1 1 1) and (2 0 0) of FCC copper nano powder in agreement with JCPDS 04-0836^33^. Some impure peaks from capping nanocomposites with pomegranate peels were detected superimposed on the broad, amorphous band of the chitosan matrix^34^ observed at 2θ = 25.8°, 28.5°, 40.5° and 49.47° (JCPDS 77-2176 and 87–0730), which revealed the presence of the K_2_O and K_2_CO_3_; while the peaks at 2θ = 30.3°, 39.4°, 47.5, 32.54, and 53.03 (JCPDS 47−1743 and 37−1497) were attributed to CaO and CaCO_3_, and the peaks at 2θ = 25.1°, 33.0°, 42.05°, 50.3°, and 51.40° were attributed to SiO_2_, Fe_2_O_3_, P_2_O_5_, carbon, and sulfur (JCPDS 41−1413, 33-0664, 5-0488, 75−1621 and 34-0941). Another peak was also observed at 2θ = 17.92º due to metal hydroxides.

**Figure 3:**
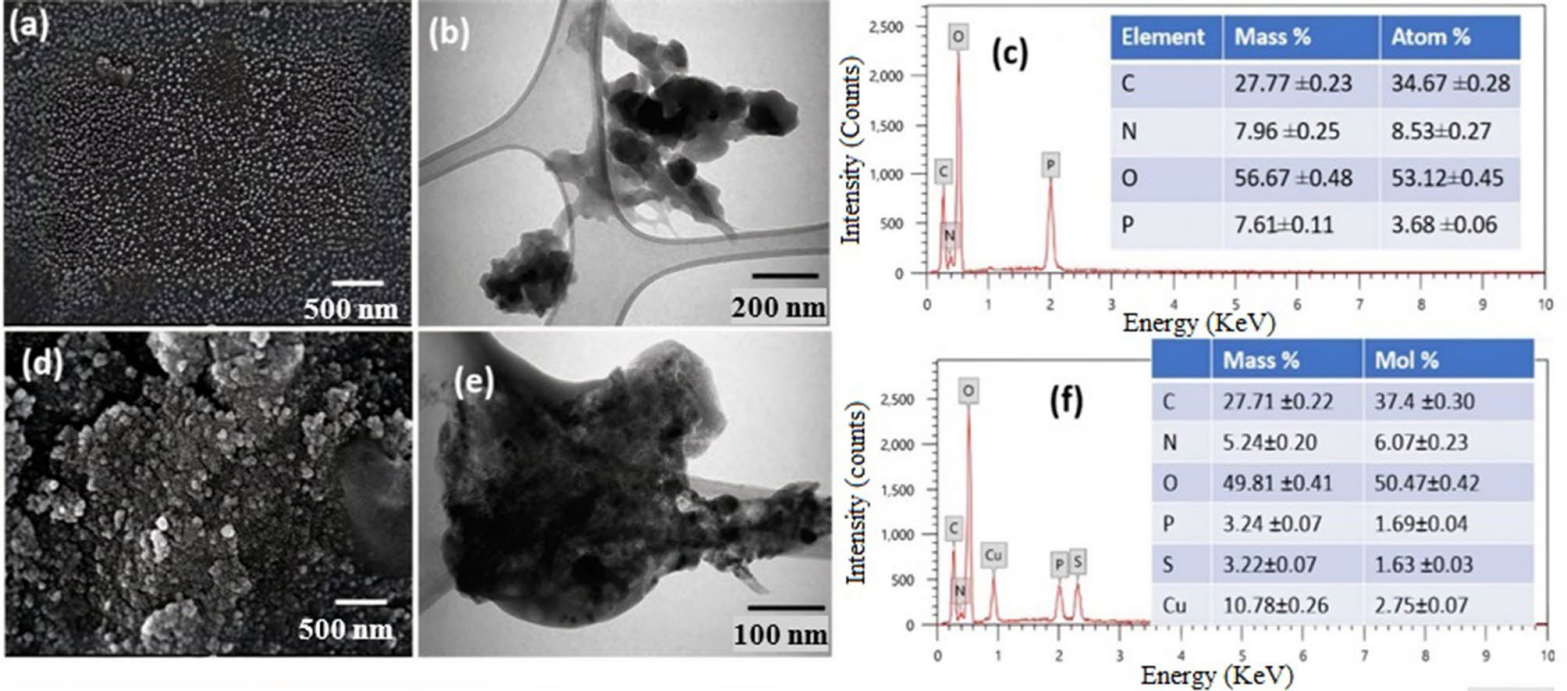
SEM images of **(a)** synthesized chitosan NPs, **(d)** chitosan–CuO nanocomposite; TEM images **(b)** synthesized chitosan NPs, **(e)** chitosan–CuO nanocomposite, and edx elemental % **(c)** synthesized chitosan NPs, **(f)** chitosan–CuO nanocomposite. This figure was created using microsoft power point 10.

Surface morphology, size, and fundamental structure of synthesized Cs-NPs and Cs–CuO nanocomposite were analyzed using SEM, TEM, and EDX. Figure 3a,b revealed a clustered, homogenous distribution of an ideal spherical shape of nanoparticles with narrow particle size distribution ranging from 20 to 30 nm. Figure 3c confirmed that these nanoparticles mainly composed of C, N, O, and P. The SEM image (Fig. 3d) showed two types of nanoparticles aggregate on the surface with particle sizes ranging from 18 to 40 nm. Figure 3f confirmed that these spherical shape particles are CuO NPs embedded on a chitosan matrix, as shown in Fig. 3e.

### Antimicrobial test

**Figure 4:**
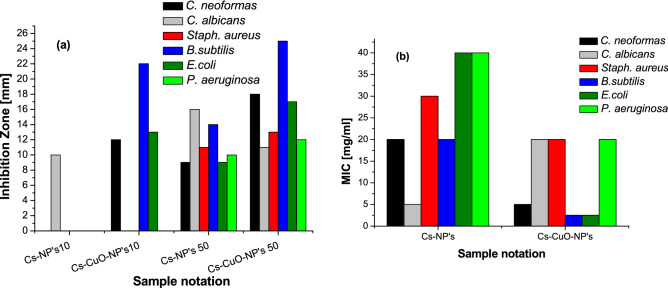
**(a)** Inhibition zone of synthesized nanoparticles of Cs-NP’s and Cs–CuO-NP’s at 10,000 and 50,000 mg/L, **(b)** minimum Inhibition Concentrations of Cs-NP’s and Cs–CuO-NP’s.

The inhibition zone assay investigated the antimicrobial activity of the Cs-NP’s and the Cs–CuO-NP’s against fungus [*Cryptococcus neoformans* (*C. neoformans*) and *Candida albicans* (*C. Albicans*)], gram-positive [*Staphylococcus aureus* (*S. aureus),* and *Bacillus subtilis (B. subtilis*)] and gram-negative bacteria [*Escherichia coli* (*E. coli)* and *Pseudomonas aeruginosa (P. aeruginosa*)], respectively. Although both synthesized samples exhibited a wide range of antimicrobial activity, the biosynthesized Cs–CuO-NP’s are expected to possess higher antimicrobial sensitivity than Cs-NP's due to the synergistic effect of Chitosan CuO and pomegranate peel extract. Two significant observations are clear from the results in Fig. 4. First, the concentration of both samples examined (10,000 mg/L and 50,000 mg/L) affects the diameter of inhibition zones growth and their antimicrobial efficiency. Second, the growth inhibition zone's diameter increases upon loading of CuO-NP's and due to the capping effect of the green extract used in the preparation of the hybrid composite. It was found that all microorganisms tested could grow under the 10,000 mg/L of Chitosan NP's except *C. neoformans* which was affected by Cs-NP's. In contrast, the similar concentration of chitosan/CuO nanocomposites inhibits the growth of *C. neoformans*, *B. subtilis*, and *E*. *coli* with diameter 22 mm, 13 mm, and 10 mm, respectively. The inhibition zone size varied according to the type of bacteria and the differences in the cell membrane structure of the three types of bacteria examined. Upon increasing the concentration of Cs–CuO-NP’s (50,000 mg/L), it implied proficient inhibition in the growth of more species, namely *Staphylococcus aureus, Pseudomonas aeruginosa*, and *Candida albicans* with inhibition zone values of 13 mm, 12 mm, and 11 mm respectively: these are considered higher values compared to the values recorded in the literature^35^.

It is well established in the literature that chitosan derivatives have been significantly inhibiting the gram-positive bacteria^36^, while copper oxide NP showed greater activity against gram-negative microorganisms, consistent with this study's findings.

Pomegranate peel extract, which acts as the capping agent (confirmed by XRD), was not randomly selected but, its high tannins and polyphenolic content has been reported as the key factors for the peel antimicrobial activity. The pomegranate peel extract showed a potent sensitivity towards Gram-positive bacteria^37^, which is similar to our results; *B. subtilis* was more sensitive than *S. aureus*, followed by *E. coli*^38^ as it could affect the transport of substrates into cell^39^. Additionally, pomegranate peel extract has significant fungal inhibitory activity. Thus, Cs–CuO-NP’s were successfully tailored to merge the activity of chitosan nanoparticles, CuO, and pomegranate peel capping extract to obtain a broad spectrum antimicrobial novel composite. The nanoparticles' activity is usually ascribed to their small size, enabling them to permeate through the bacterial cell membrane^40^. Besides, the positively charged hybrid Cs–CuO-NP’s could block the cells' nutrient intake due to their interaction with negatively charged lipidic bacterial membrane, thus reducing both cell growth and viability^41^. It is also worth noting that the efficient antibacterial activity of hybrid Cs–CuO-NP’s could be due to reactive oxygen species generation by the nanoparticles attached to the bacterial cells, which in turn provoked an enhancement of intracellular oxidative stress^42^. The presence of CuO nanocrystals in Cs–CuO-NP’s improves the antibacterial activity by releasing and diffusing Cu^2+^ ions in the agar medium. These Cu^2+^ ions induce the production of reactive oxygen species (ROS) such as HO^•−^, O_2_^•2−^, HO_2_^•−^ and H_2_O_2_, which cause cell integrity when interacting with the bacteria cells^43^.

Minimum inhibitory concentration MIC is a quantitative method used to analyze antibacterial activity. In the current work, MIC was applied to check the two synthesized samples' antibacterial and antifungal activity. The recorded MIC values in Fig. 4b support the inhibition test zone results, which shows an enhanced activity of the Cs–CuO-NP's towards the gram-negative bacterial strain and the gram-positive bacterial strain compared to Cs-NP’s, which was consistent with earlier research^41^.

### Hematological test

Undoubtedly that RBCs and platelets play an essential role in both thrombosis and hemostasis. RBCs affect the Rheological blood viscosity and platelet aggregation, enabling them to act as a procoagulant and prothrombotic blood component. RBC’s interact with platelets, endothelial cells, and fibrinogen, which in turn leads to their incorporation into the thrombin.

**Figure 5:**
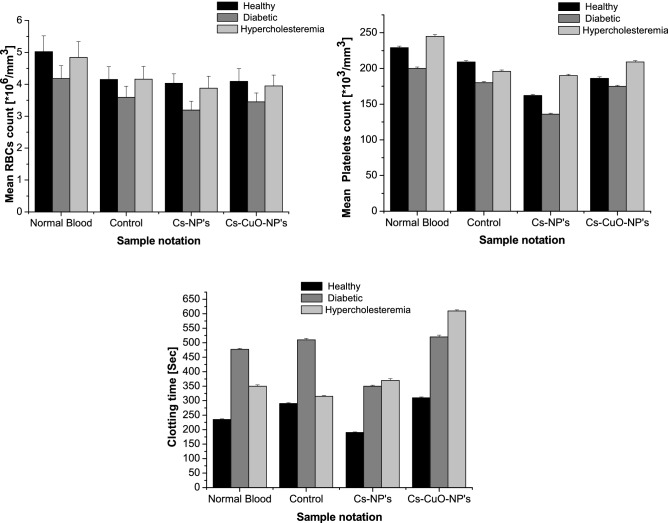
The effect Cs-NP's and Cs–CuO-NP's nanocomposite on **(a)** mean RBC's count, **(b)** mean platelets count and clotting time after adding to healthy, diabetic and hypercholesteremia blood samples.

In comparison with control blood samples, a noticeable decrease in mean RBCs and platelet counts was observed by adding Cs-NP's to the blood rather than adding Cs–CuO-NP's as shown in Fig. 5a,b. Figure 5a shows that adding Cs-NP's decreases the mean RBC count by 2.8%, 11.1%, and 6.7% in healthy, diabetic, and hypercholesteremia blood samples, respectively. The same decreasing pattern was observed, which is determined to be 1.44%, 3.8%, and 5.0% when adding Cs–CuO-NP's into healthy, diabetic, and hypercholesteremia blood samples, respectively. However, adding Cs-NP's leads to a decrease in mean platelets count to 22.4%, 24.4%, and 3.0% in healthy, diabetic, and hypercholesteremia blood samples, respectively, in comparison to 11.0% and 2.7% lesser in platelets count upon adding Cs–CuO-NP's into healthy and diabetic blood samples, respectively. Simultaneously, it increases the platelet count in hypercholesteremia blood samples (Fig. 5b).

The effects of Cs-NP’s and Cs–CuO-NP’s on the coagulation time of healthy, diabetic, and hypercholesteremia blood samples in vitro were also investigated (Fig. 5c). It was shown in Fig. 5c that Cs-NP's can decrease clotting time for healthy and diabetic blood samples. An opposite effect was observed in hypercholesteremia blood samples while adding CuO capped with *P. granaum* extract to Cs-NP’s (synthesized nanocomposite) act as anti-coagulant increasing clotting time.

The chitosan nanoparticle (Cs-NP’s) gains hemostatic properties from its net positive charge, which depends on the DD and number of pronated amine groups^44^. These amine groups initiate attraction with negatively charged red blood cells and platelets (Fig. 5a,b), enabling Chitosan to build a mesh-like spatial structure, which promoted interaction between chitosan and blood components, facilitating the formation of blood clotting. Also, Cs-NP's is able to gradually depolymerized to release *N*-acetyl-d-glucosamine, which is transported to cells via glucose receptors and has a role in protecting against atherosclerosis. *N*-acetyl-d-glucosamine initiates fibroblast proliferation, aids in providing collagen deposition orders, and stimulates the increased synthesis of natural hyaluronic acid levels at wound sites. It was proved in a previous study that Chitosan with moderate DD nearly 68.36% had the most significant procoagulant effect^45,46^. This is attributed to a higher degree of DD had more amino groups and hydroxyl groups in the molecules, which form a stronger hydrogen bond inside the molecules, leading to a crystalline structure of Chitosan that could hardly interact with blood components to promote coagulation^45^.

Adding Copper oxide nanoparticles (nCuO) to Cs-NP’s plays a vital role in masking and inhibiting the inflammatory activity of Chitosan in addition to enhancing wound healing properties of chitosan^47^. It was proved histologically that nCu could stimulate proliferation and migration of fibroblasts. Some Copper dependent enzymes help in the synthesis of collagen to facilitate wound healing. It was clearly known that Chitosan is polycationic at acidic media, so it chelates metallic ions such as Fe, Cu, or Mg^48^. This proves that Cu ions chelate chitosan nanoparticles suppressing sites of interaction with RBCs and platelets. This could account for the increasing RBCs and platelet count in Fig. 5a,b.

On the other side, when comparing the results of Cs-NP’s with Cs–CuO-NP’s, it was observed that adding Cs–CuO-NP’s lead to more RBCs and platelets and clotting time (Fig. 5), this is due to the presence of *P. granatum* extract as a capping agent for synthesized composite. It was suggested in previous work that the presence of *P. granatum* inhibits platelet aggregation due to the presence of anthocyanidins in *P. granatum* that are responsible for suppressing cyclooxygenase^49^ or may be due to the decrease in fibrinogen level^50^. Increasing clotting time is due to the anti-coagulant effect of *P. granatum*, which inhibits thrombin and intrinsic coagulation factor^51^.

From the previous results, it could be concluded that at Cs-NP's are hemostats; they can act as prothrombin or procoagulant while Cs–CuO-NP's are recommended anti-coagulant.

## Conclusion

The main points concluded from this work could be summarized as the following, *Point(I)* the characterization of biogenic synthesized Cs–CuO-NP's and Cs-NP's found that both nanoparticles are in a spherical shape with particles size around 20–40 nm. The characterization also provides Cs–CuO-NP's formation as hybrid nano–composite from nanochitosan and copper oxide capped with extract of pomegranate peel. *Point(II)*, the antimicrobial activity inhibition zone test for both Cs-NP's and Cs–CuO-NP's show the great ability of Cs–CuO-NP's as antimicrobial agent comparing to Cs-NP's . These results clarify that Cs–CuO-NP's is highly sensitive to *C. neoformas*, *B. subtilis*, and *E. coli* at a concentration (10,000 mg/L), while the same concentration (10,000 mg/L) of Cs-NP's against all microorganisms, under examination, was effected only on *C. neoformas*. On the other hand, increasing the concentration of both Cs-NP's and Cs–CuO-NP's to 50,000 mg/L increases the sensitivity of Cs-NP's as an antimicrobial agent and increases the ability of Cs–CuO-NP's by high migraine enough to be lethal for all microorganisms under investigation. The MIC test also explains the role of the hybrid composite Cs–CuO-NP's as antimicrobial, which found lethal for all microorganisms under test in the range of concentration below 20,000 mg/L Cs–CuO-NP's, and Cs-NP's found affected only after 20,000 mg/L at least for all microorganism except for *C.albicans* species, it was found lethal at 5,000 mg/L. This comparison between both biogenic nanoparticles proves the importance of a hybrid composite of Copper and capping agent with nano chitosan in enhances the antimicrobial properties of any biogenic nanoparticles. *Part (III)*, the hematological activity test for both Cs-NP's and Cs–CuO- NP's proved that Cs-NP's particles are hemostat, acts as prothrombin or procoagulant activator used to accelerate the blood clotting process for a healthy and diabetic patient to prevent Scar. While Cs–CuO-NP's act as anti-coagulant could be used as a coating for a coronary stent or drug delivery to prevent arteriosclerosis.

## Materials and methods

### Chemical materials

Chitosan has been purchased from ChemCruz (75% deacetylation), copper sulphate penta hydrate from Techno pharm chem, Glacial acetic acid (99.5%), and TPP (sodium tripolyphosphate anhydrous) from Loba chemie. Finally, pomegranate peel from the Egyptian market.

### Nanoparticles synthesis

#### Preparation of the green extract

A mass of 40 g of pomegranate peel (*Punica granatum*) powder is added into 1L of distilled water. The mixture is boiled for 30 min, followed by filtration to obtain a clear filtrate. This clear filtrate is kept in the fridge at 4 °C and is considered as the plant's extract^28^.

#### Synthesis of chitosan nanoparticles

The nano-chitosan has been prepared by the ionic gelation method^52^, where 0.5 g chitosan (75% deacetylation) was dissolved in 50 mL of 1.0% (v/v) acetic acid. Afterward, 1.0% (w/v) of the trisodium polyphosphate (TPP) was added to the former solution with constant stirring for 1 h. The produced white precipitate (nano chitosan) was isolated and washed several times with deionized water. Finally, the product was dried in an oven overnight at 60 °C.

#### Bio-synthesis of chitosan–copper oxide nanoparticles

Cs–CuO-NP's was synthesized through the following few simple steps. Firstly, 0.5 g of Chitosan was dissolved in 50 mL of 1.0% acetic acid followed by dropwise addition of 1.0% of TPP^52^. Secondly, 50 mL of 1.0 M hydrous copper sulfate was added to the mixture of Chitosan and TPP, followed by the dropwise addition of 50 mL of a plant extract with constant stirring and heating at 80 °C for 1 h. Finally, the resulting nanoparticles were isolated by decantation, washed several times with deionized water, and dried in an oven at 80 °C.

### Characterization of synthesised nanoparticles

#### Structural characterizations

Synthesized nanoparticles were examined via Fourier Transform Infrared (FTIR) spectra to investigate the presence of functional and characteristic groups using a Shimadzu FTIR spectrophotometer. The spectra were carried out at a resolution of 4.0 cm^−1^. To obtain a reasonable signal-to-noise ratio, 64 scans were completed. The dried nanoparticles were pressed with KBr and tested^53^. To identify the specific bands of the Cs-NP's and CuO-NP's, an x-ray powder diffractometer using Shimadzu XRD with Cu Kα radiation (λ = 1.5418 Å) at a scanning speed of 0.2 S, was used.

#### Morphological characterizations

The morphology and the elemental analysis of the synthesized nanoparticles were performed using SEM (FEI, ISPECT S50, and Czech Republic). SEM was operated at 20 kV with a working distance of around 10 mm. The samples were fixed on a metallic stub with double-sided adhesive tape. Images were taken at different magnifications to obtain a better visual inspection and noting specimens' important features. For TEM, the synthesized nanoparticles were dispersed in ethanol under sonication for 5 min and deposited onto TEM grids with carbon support film. TEM grids were mounted into the TEM upon evaporation of water in the air at room temperature. The specimens' images were recorded using TEM, FEI, Morgagni 268, and Czech Republic at 80 kV. Finally, the EDAX analysis were performed using EDX-8000 and, Shimadzu^54,55^.

### Antimicrobial test

Antimicrobial activities of Cs-NP's and Cs–CuO nanocomposites were carried out according to NCCLS recommendations (National Committee for Clinical Laboratory Standards, 1993). Inhibition zone primary screening tests were performed by the well diffusion method^56^. Inoculums suspension was prepared using the tested organisms colonies grown overnight on an agar plate. Chitosan nanoparticles and synthesized nanocomposites were dissolved in DMSO with different concentrations (50,000, 10,000, 5000, and 2500 mg/L). The diameter of the inhibition zone indicating antimicrobial activities was measured after 24 h incubation at 37 °C. This study investigated Cs-NP’s and Cs–CuO-nanocomposites against fungi [*Cryptococcus neoformans* (*C. neoformans)* and *Candida albicans (C. albicans*)], gram-positive [*Staphylococcus aureus* (*S. aureus)* and *Bacillus subtilis (B. subtilis*)] and gram-negative bacteria [*Escherichia coli* (*E. coli)* and *Pseudomonas aeruginosa (P. aeruginosa*)].

### Hematological test

#### Chitosan solution preparation

10,000 mg/L of Cs-NP’s and Cs–CuO nanocomposites were dissolved in 1% acetic acid separately for 2 h, stirring at room temperature.

#### Blood collection

10 mL of whole healthy, diabetic, and hypercholesteremia blood was collected from the antecubital vein using 21-gauge needles with three-way stop-cocks to minimize tourniquet pressure. The collected blood was aliquoted into three tubes containing 3.8% of sodium citrate anti-coagulant tubes for all the studies except for blood coagulation. The subject selection was conditional on normal platelet counts for healthy blood, high blood glucose ranged from 180 to 200 mg/dL for diabetic blood, and LDL cholesterol ranged from 160 to 180 mg/dL for hypercholesterolic blood.

Ethical approval for the research was obtained by the Institutional Review Board of Imam Abdulrahman Bin Faisal, Kingdom of Saudi Arabia (Reference number: IRB-2020-03-339). This research was performed under the Declaration of Protecting Human Research Participants Online Training (PHRP no. 2852904) involving Human Subjects, and all participants signed an informed consent form.

#### Complete blood count (CBC)

CBC was carried out on the hematology analyzer CELLTAC to determine hemoglobin level (HGB, g/L), the red blood cell count (RBC, count/mm^3^), and platelet counts (PLT, count/mm^3^). CBC was measured by adding 0.5 mL of Cs-NP’s and Cs–CuO nanocomposite solutions into 1.5 mL of each blood sample, aliquoted in 3.8% sodium citrate anti-coagulant tubes. The blood incubated in a water bath at 37 °C for 5 min, and it was then measured using CELLTAC.

#### Blood coagulation time (BCT)

BCT was measured by adding a solution of 0.5 mL of Cs-NP's and Cs–CuO nanocomposite into 1.5 mL from each blood sample. The blood was incubated in a water bath at 37 °C for 5 min, and then the blood coagulation was observed by inclining the tube at 30-s intervals until the blood is clotted. When the blood flow was not observed up on the tube's inclination at 90º angle, which indicated blood became coagulant. BCT was measured immediately after blood collection until blood coagulation was observed.

### Statistical analysis

Data were analyzed using SPSS 11 program. Numerical values were presented as means ± SD.
